# Kidney Transplantation from an extracorporeal membrane oxygenation-supported brain-dead donor: A case report

**DOI:** 10.1097/MD.0000000000011106

**Published:** 2018-06-29

**Authors:** Pei-Jhang Chiang, Shou-Hung Tang, Chiao-Ching Li, Meng-Han Chou, Yu-Chun Lin, Sheng-Tang Wu

**Affiliations:** a Division of Urology, Department of Surgery; b Department of Pathology, Tri-Service General Hospital, National Defense Medical Center, Nei-Hu, Taipei, Taiwan.

**Keywords:** brain-dead donor, extracorporeal membrane oxygenation, kidney transplantation, pretransplant biopsy

## Abstract

**Rationale:**

Extracorporeal membrane oxygenation (ECMO) can deliver effective respiratory and circulatory maintenance to organ donors, improve organ function, and shorten warm ischemic time before harvesting. However, ECMO-supported brain-dead donors (DBDs) still have a high risk of acute kidney injury related to decreased renal oxygen delivery and inflammatory damage, which may cause early graft failure.

**Patient concerns:**

Kidney transplantation from an ECMO-supported DBD.

**Diagnoses:**

We found an extremely abnormal “very dark blue” appearance of the graft kidneys from an ECMO-supported DBD during kidney procurement.

**Interventions:**

Rather than discarding the graft kidneys, we performed an on-table biopsy. Pretransplant biopsy results revealed minimal interstitial fibrosis in the section of these graft kidneys.

**Outcomes:**

Two candidates received graft kidneys, and the two grafts remained functional until the 8-month follow-up.

**Lessons:**

Currently, there is no standard method for evaluating graft kidney function of ECMO-supported DBDs. Regardless of the donors’ preoperative serum creatinine (SCr) level, estimated glomerular filtration rate (eGFR), or gross appearance of the graft kidney, we believe that it is more reliable to include pretransplant biopsy as a criterion in clinical practice to safely accept kidneys from ECMO-supported DBDs.

## 1 Introduction

Extracorporeal membrane oxygenation (ECMO) can deliver effective respiratory and circulatory maintenance to organ donors, improve organ function, and shorten warm ischemia time before harvesting. Compared with kidney transplant using non-ECMO organs, kidney transplant using ECMO-supported brain-dead donors (DBDs) does not appear to have a higher rate of graft dysfunction.^[1,2]^

However, kidneys from ECMO-supported DBDs are still at a high risk of acute kidney injury (AKI) related to decreased renal oxygen delivery to inflammatory damage^[3,4]^ and may be a cause of early graft failure. Currently, there is no standard evaluation criteria to assess such graft kidneys. Herein, we report our experience that pretransplant biopsy could be more important than gross appearance, serum creatinine (SCr) level, or estimated glomerular filtration rate (eGFR) to safely accept kidneys from ECMO-supported DBDs.

## 2 Case report

### 2.1 Patient information

The donor, a 39-year-old male with a history of schizophrenia, fell from a fifth floor window and was taken to Tri-Service General Hospital, Taipei, Taiwan. Computed tomography scan revealed multiple rib fractures, pelvic fracture with active bleeding, and dissection of the descending aorta with intramural hematoma. Consequently, the patient underwent bilateral anterior thoracotomies and transcatheter embolization of the pelvic vessels for acute resuscitation.

After 14 days, the patient underwent thoracic endovascular aortic repair of the descending aortic aneurysm. During the procedure, the patient experienced oxygen desaturation and cardiac arrest, and advanced cardiac life support was performed immediately. After return of spontaneous circulation, ECMO was set up due to persistent bradycardia and poor tissue perfusion, possibly caused by pulmonary embolism.

Hypoxic-ischemic encephalopathy was not improved after 1 week; therefore, the diagnosis of brain death was made by 2 senior doctors, and the family decided to enter the critical hospice pathway and donate his organs.

### 2.2 Clinical findings

**Figure 1:**
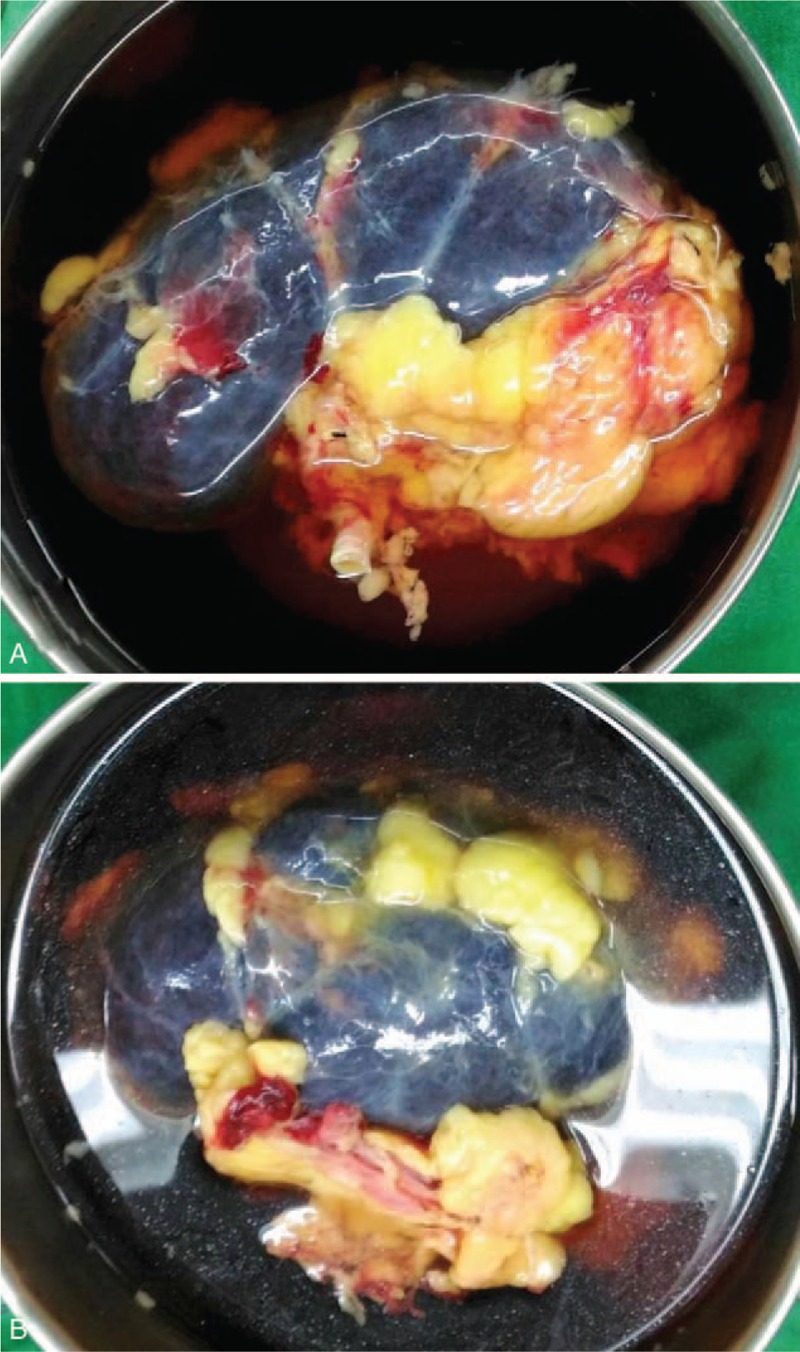
The donor kidneys are “very dark blue” in color. Right graft kidney (A) and left graft kidney (B).

**Figure 2:**
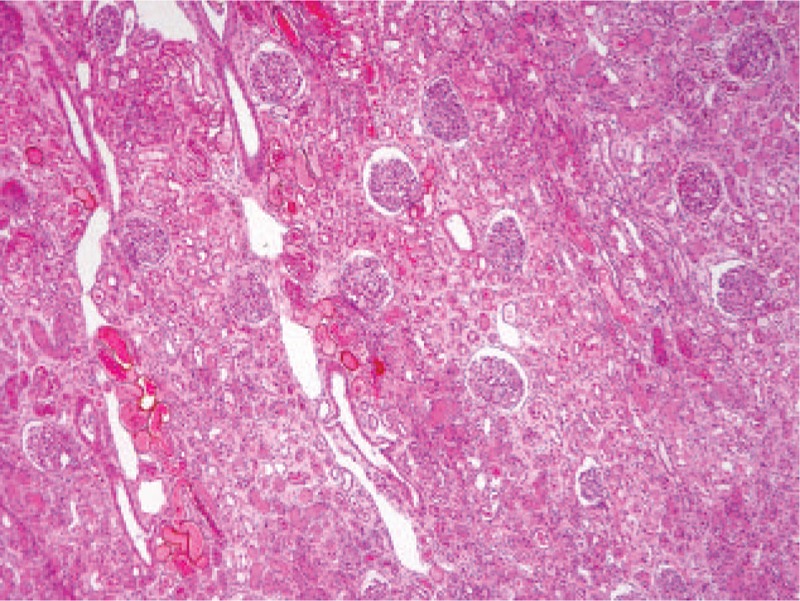
Hematoxylin and eosin micrograph of a histological section of the donor kidney showing intact glomeruli and congestion with lymphocyte infiltration.

Organ donation was made at 21 days after the patient's trauma (7 days after the ECMO was placed). The patient's initial SCr on admission was 0.9 mg/dL and rose to 4.7 mg/dL at organ harvesting. During kidney procurement, we discovered an extremely abnormal appearance of the kidneys, which were “very dark blue” in color (Fig. 1). Rather than discarding the kidneys, we performed an on-table biopsy. Microscopically, this section showed only minimal interstitial fibrosis of parenchyma. The glomeruli, tubules, and vessels were intact (Fig. 2). The final grade of pretransplant biopsy based on the Remuzzi Score System was 1 of 12, indicating single kidney transplantation.

After informed consent, 2 candidates received grafts; one was a 47-year-old female with lupus nephritis who developed end-stage renal disease (ESRD) and underwent hemodialysis and the other was a 58-year-old female with immunoglobulin A nephropathy leading to ESRD who underwent peritoneal dialysis (Table 1). Both recipients received dialysis within the first postoperative week due to delayed graft function.

**Table 1:**
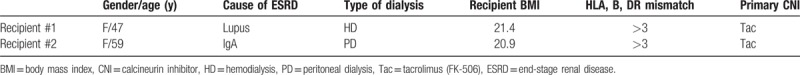
Demographic data of recipients.

### 2.3 Follow-up and outcomes

With the use of immunosuppressive therapy, the SCr of these 2 patients decreased from 8.5 and 17.6 mg/dL to 1.1 and 1.8 mg/dL, respectively, at the 1-month postoperative follow-up, and these 2 grafts remained functional at the 8-month follow-up (Table 2).

**Table 2:**
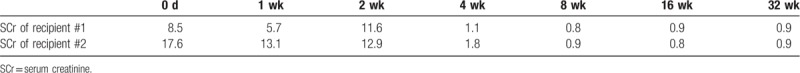
Serial serum creatinine levels of the 2 recipients after kidney transplantation.

Written informed consent to publish the case report was provided by the patients, and the consent procedure was approved by the Ethics Committee of Tri-Service General Hospital.

## 3 Discussion

The shortage of kidneys from cadaveric donors is a global problem. As a bridge to brain death declaration, the concept of using ECMO to expand the donor pool has become widely accepted. Despite the advantage of maintaining organ perfusion, using ECMO also carries the risk of decreased renal oxygen delivery and AKI.^[3]^ High incidence of AKI in ECMO-supported patients has been reported in several studies.^[5,6]^

There are various protocols for estimating the quality of a graft kidney. For conventional deceased or living donor kidneys, Remuzzi et al^[7]^ have described the global kidney score based on pretransplant donor biopsy, while other systems have used SCr, eGFR, or macroscopic anatomy in combination with clinico-histopathological parameters to streamline and determine the allocation of kidneys that otherwise would have been discarded.^[8–12]^

To our knowledge, there is no standard method for evaluating kidneys from ECMO-supported DBDs. Because ECMO activates an acute inflammation-like response and leads to acute tubular necrosis within 48 hours,^[4]^ physicians may get confused whether the graft kidneys should be allocated based on pre- or post-ECMO condition.

In a recent study, SCr or eGFR level of ECMO-supported DBDs did not seem relevant to kidney graft survival.^[1]^ In general, the macroscopic appearance indirectly reflects graft health, especially when acute tubular necrosis or infarction occurs. However, in this case, histopathological examinations have proven to be more reliable and precise with respect to graft function and survival. This case report suggests that preoperative evaluation and intraoperative frozen section consultation play a more important role in ECMO-supported donation than previously thought.

## 4 Conclusions

Using ECMO maintains organ perfusion, and ECMO-supported kidney donors expand the donor pool, but the risk for AKI related to decreased renal oxygen delivery and inflammatory damage might be a cause of early graft failure.

In summary, rather than SCr, eGFR, or macroscopic anatomy, we believe that it is more beneficial to include pretransplant biopsy as a criterion in clinical practice to safely accept kidneys from ECMO-supported DBDs. Moreover, long-term follow-up is required to confirm the benefit of this practice.

## Author contributions

**Conceptualization:** Sheng-Tang Wu.

**Data curation:** Chiao-Ching Li, Meng-Han Chou, Yu-Chun Lin.

**Supervision:** Sheng-Tang Wu.

**Writing – original draft:** Pei-Jhang Chiang.

**Writing – review & editing:** Shou-Hung Tang.
